# Virchow ne meurt jamais

**DOI:** 10.11604/pamj.2015.22.29.7644

**Published:** 2015-09-14

**Authors:** Youssef Kort, Naziha Khammassi

**Affiliations:** 1 Faculté de Médecine de Tunis, Service de Médecine Interne, Hôpital Razi, La Manouba 2010, Tunisie

**Keywords:** Tumeur osseuse, complication osseuse, compression de tissus mous, bone tumor, bone complication, compression of soft tissue

## Image en medicine

Les exostoses représentent la forme la plus fréquente des tumeurs osseuses bénignes, elles peuvent être uniques ou multiples se formant au niveau des métaphyses des os longs avec une prédilection pour le fémur et l'humérus. Elles sont le plus souvent asymptomatiques mais peuvent parfois être révélées par une complication osseuse ou à l'occasion de la compression de tissus mous de voisinage. Les compressions vasculaires sont plus rares. Patient âgé de 45 ans était hospitalisé pour un bilan étiologique d'une embolie pulmonaire. L’échographie doppler veineuse des membres inférieurs concluait à une thrombose veineuse profonde. Un bilan étiologique exhaustif était sans anomalies. L'examen clinique était normal en dehors d'une masse dure d'environ 10cm de grand axe du mollet gauche avec diminution du ballottement. La radiographie des jambes notait une excroissance osseuse ovalaire et bien limitée de la partie proximale du péroné gauche. Plusieurs diagnostics ont été évoqués notamment un chondrome sous périosté vieilli, un sarcome parostéal et une dysplasie épiphysaire hémimélique. Les radiographies du bassin et des fémurs étaient sans anomalies. L’échographie des parties molles confirmait la nature osseuse de cette excroissance et excluait une anomalie musculaire associée. Les caractéristiques radiologiques de la masse à savoir une néoformation osseuse dont la corticale est en continuité avec la corticale de l'os sous jacent était en faveur d'une exostose ou ostéochondrome. Etant donné le caractère symptomatique de cette exostose, une exérèse chirurgicale a été indiquée.

**Figure 1:**
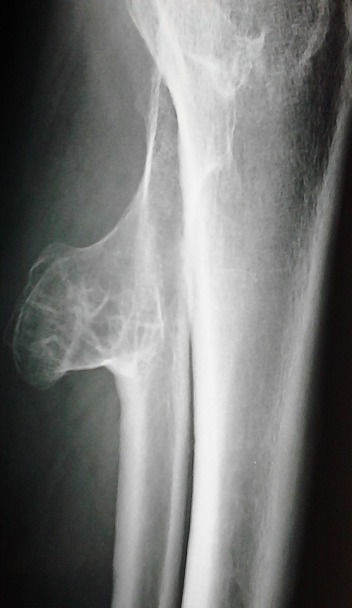
Excroissance osseuse ovalaire et bien limitée de la partie proximale du péroné gauche

